# Bile Acids in Cardiovascular Diseases: Don’t Forget Hyocholic Acid

**DOI:** 10.14336/AD.2023.0603

**Published:** 2024-02-01

**Authors:** Piercarlo Minoretti, Enzo Emanuele

**Affiliations:** ^1^Studio Minoretti, Oggiono (LC), Italy; ^2^2E Science, Via Monte Grappa, 13, I-27038 Robbio (PV), Italy

**To Editor,**

We have read with interest the extensive review by Zhang et al. [1], in which the authors delve into the involvement of bile acids (BAs) in the development of cardiovascular diseases (CVDs). The authors deserve praise for their forward-thinking perspectives on the pharmacological potential of certain BAs. For instance, they highlight the protective role of ursodeoxycholic acid in CVDs, as well as the promise shown by synthetic BAs analogs like obeticholic acid and colesevelam [1]. Nonetheless, it is regrettable that the authors overlooked the potential significance of hyocholic acid (HCA) as a biomarker and therapeutic target for CVDs, particularly those arising in the context of metabolic disorders, in their discussion of future research directions.

HCA (3α, 6α, 7α-trihydroxy-5β-cholanoic acid) is a minor component of the human BAs pool, characterized by one of the lowest hydrophobicity indices [2]. Interestingly, it accounts for approximately 76% of the BAs pool in pigs, a species known for its exceptional resistance to diabetes [2]. In recent years, growing evidence has accrued that HCA may play a role in the development of human metabolic disorders. A study by Zheng et al. [2] demonstrated that HCA administration in diabetic mouse models significantly improved serum fasting glucagon-like peptide-1 (GLP-1) secretion and glucose homeostasis, outperforming taurourso-deoxycholic acid. In addition, HCA was found to upregulate GLP-1 production and secretion in enteroendocrine cells by simultaneously activating the G-protein-coupled bile acid receptor, TGR5, and inhibiting the farnesoid X receptor [2]. This unique mechanism distinguishes HCA from other BAs species. The same research team showed that both obesity and diabetes are associated with lower serum concentrations of HCA species in humans [3]. Moreover, individuals with pre-diabetes tend to have lower levels of HCA in their feces. Remarkably, serum HCA levels increase in patients after undergoing gastric bypass surgery and can even predict diabetes remission two years post-surgery [3]. These findings were replicated in two independent, prospective cohorts, where serum HCA species were identified as strong predictors for metabolic disorders in 5 and 10-year follow-ups, respectively [3]. More recently, Xu and colleagues [4] reported that decreased HCA levels induced elevated blood glucose in a transgenic porcine model of metabolic disease.

Considering the emerging significance of HCA as a pivotal mediator of metabolic diseases in humans, and the firmly established connection between metabolic disorders and CVDs, it is of paramount importance to explore HCA’s role concerning the presence and severity of CVDs in clinical cohorts. This investigation should encompass individuals with and without metabolic abnormalities, ensuring a holistic understanding of HCA’s influence on human cardiovascular health. Consequently, this will lay the groundwork for HCA’s potential as a therapeutic target in the treatment and prevention of CVDs.

Unfortunately, HCA continues to be a neglected player in the context of metabolic diseases and cardiovascular health. Several factors contribute to this oversight, such as its low concentrations in the BAs pool and the lack of a cost-effective, easily accessible immunoassay kit for HCA measurement. Therefore, the initial step towards unlocking HCA’s clinical potential involves establishing an affordable, straightforward assay to determine its levels in serum and fecal samples. This advancement would enable HCA assessments in a wider variety of clinical and research contexts. Once a dependable assay becomes available, researchers can investigate different methods to increase HCA levels in the human BAs pool. Potential strategies may encompass dietary interventions, pharmacological agents, or even targeted microbiome modulation. By comprehending how to effectively elevate HCA levels, we can initiate the evaluation of its therapeutic potential in preventing and treating metabolic disorders and CVDs.
